# Multiple pressures and vegetation conditions shape the spatiotemporal variations of ecosystem services in the Qinghai-Tibet Plateau

**DOI:** 10.3389/fpls.2023.1127808

**Published:** 2023-01-19

**Authors:** Yuanxin Liu, Yihe Lü, Mingyue Zhao, Bojie Fu

**Affiliations:** ^1^ State Key Laboratory of Urban and Regional Ecology, Research Center for Eco-Environmental Sciences, Chinese Academy of Sciences, Beijing, China; ^2^ Academy for Multidisciplinary Studies, Capital Normal University, Beijing, China; ^3^ University of Chinese Academy of Sciences, Beijing, China; ^4^ Institute of Environment and Sustainable Development in Agriculture, Chinese Academy of Agricultural Sciences, Beijing, China

**Keywords:** ecosystem services, environmental change, bundle analysis, structural equation model, vegetation condition, human activities

## Abstract

Human activities and environmental change can impact the supply of ecosystem services (ESs) as pressures. Understanding the mechanisms of these impacts is crucial to support ecological conservation and restoration policy and applications. In this study, we highlighted the contribution of vegetation to mitigating these impacts on ESs in the Qinghai-Tibet Plateau (QTP) of China. First, we identified hot and cold spots of pressures from human activities and environmental factors and mapped the cumulative provision of five ESs (i.e., water yield, soil retention, carbon sequestration, habitat quality, and landscape aesthetics). Then, we clustered these ESs into five bundles based on their supply level. Furthermore, structural equation modeling was used to quantify the pathways of multiple pressures on ESs. The results indicated that 1) for 2000, 2010 and 2019, the percentages of hot spots with high pressure were 28.88%, 27.59% and 45.66% respectively, with significant spatial heterogeneity from northwest to southeast; 2) both regions with high and low cumulative ES values experienced increased volatility; and 3) the joint effects of multiple pressures shaped ESs through pressure-ES (direct) and pressure-vegetation-ES (indirect) pathways. Specifically, precipitation had the largest positive effect on regulating services (*rα* ≥ 0.76), and landscape fragmentation had the largest negative effect on cultural services (-0.10 ≤ *rα* ≤ -0.07). Vegetation played an important role in modulating multiple pressures on ESs. This study contributes to ecosystem management by effectively coping with anthropogenic and environmental pressures and sustaining the supply of ESs, particularly in alpine and plateau regions.

## 1 Introduction

Ecosystem services (ESs) are benefits or contributions provided by natural ecosystems that are available for humans (Costanza et al., 2011). There is a growing recognition that actions to conserve and improve ES provisions are urgently needed at different scales (IPBES, 2019; Meacham et al., 2022). Understanding the driving mechanism of ES change is essential for long-term ecosystem conservation (Zhang et al., 2018; Han et al., 2020). ESs are both spatially heterogeneous and interactive (Liu et al., 2019b). Many researchers have investigated the interactive relationships of multiple ESs, as well as the spatial aggregation of ESs (i.e. bundles) (Raudsepp-Hearne et al., 2010; Crouzat et al., 2015). As the relationship between nature and humans becomes increasingly apparent, it is crucial to understand the progress of human and environmental impacts on ESs to support ecological management and planning (Mandle et al., 2020).

With the increase in the intensity of human activities (e.g., urbanization) and changes in environmental factors (e.g., meteorological factors and landscape fragmentation), ESs have shown many ways of changing over time (Liu et al., 2019b; Vigl et al., 2021; Fu et al., 2022). For example, landscape pattern change inevitably leads to changes in ecosystem structure and ES provision (Wang et al., 2017). Previous studies indicated that climate change increased agricultural production in some regions because of higher temperature and CO_2_ concentration to promote crop photosynthesis, but many regulating and cultural ESs were mostly negatively affected (Braun et al., 2019; Vigl et al., 2021). A study in Central Asia demonstrated that climate change had a higher contribution to the spatial distribution of ESs than socio-economic factors (Li et al., 2021). In addition, landscape fragmentation changes the demographic and functional tree composition of species, which may translate into a loss of regulating service potential (de Avila et al., 2018). Thus, how to assess and effectively cope with multiple pressures is paramount to successfully implement ecosystem management (Lü et al., 2021; Vigl et al., 2021). Although the impact of multiple pressures on ESs has been extensively evaluated by previous studies, the existing evidence is more concentrated on direct relationship between pressures and ESs, and the results are inconsistent (Li et al., 2021; Pyles Marcela et al., 2022). Another challenge is the change in the pressure-ES relationship over time, which makes it difficult for policy-makers to conduct scientific management and optimization of ecosystems (Liu et al., 2019b; Hou et al., 2021). Most studies are snapshots in time, revealing only the short-term state of the pressure-ES relation (Meacham et al., 2022). The impact of pressures on ESs may exhibit positive or negative effects and vary over time and space (Li, 2022). The historical context of case studies will help to identify how consistent the associations between pressures and ESs are (Liu et al., 2019b; Ma et al., 2021).

While many studies have investigated various impacts of pressures on ESs (Li et al., 2021; Rijal et al., 2021; Vigl et al., 2021), few studies have examined the role of vegetation (Han et al., 2020). Vegetation is fundamental to ecosystem functioning and supports ecosystem stability under multiple pressures (Lü et al., 2012; Feng et al., 2017; Bizzaro et al., 2022). In this sense, ensuring that vegetation is in good state and resilient is very important to support human well-being (Rendon et al., 2022). Furthermore, ESs are benefits provided by ecosystems to humans, and changes in vegetation will inevitably affect ecosystems, thereby changing the supply of ESs (de Groot et al., 2010; Bizzaro et al., 2022). For example, forest cover has been proven to be correlated with provisioning and cultural ESs in a rapidly changing landscape in Nepal (Rijal et al., 2021). Li (2022) used the normalized difference vegetation index (NDVI) to analyze its relationship to various ESs and found a significant positive correlation with carbon sequestration. Additionally, pressures may alter the supply of ESs by affecting vegetation conditions, which is an essential indirect pathway that needs to be focused on (Yapp et al., 2010; Dong et al., 2022). However, the relationship between pressures, vegetation conditions and ESs is still unclear. For this, the Qinghai-Tibet Plateau (QTP), an essential ES supply pool, was selected as a typical case. We quantitatively evaluated the relationship between multiple pressures and ESs, as well as the role of vegetation.

The QTP is a water tower area and an essential eco-safety shelter in the world, as well as a focus for ecological civilization construction in China. The QTP is of great significance to the sustainable development of the region and the ecological conservation of China and the world (The State Council Information Office of the People’s Republic of China, 2018). As the world’s highest plateau, the QTP has a fragile ecosystem, which has proved to be more vulnerable to climate change (Yao et al., 2012). Additionally, the interaction of multiple pressures in the QTP also complicates the assessment of regional ESs. General atmospheric circulation models have shown that human induced land use changes in the QTP significantly affect the regional climate (Kang et al., 2010). There is insufficient research regarding the spatiotemporal variations in pressures, as well as the relationship among pressures, vegetation conditions and ESs in the QTP. At such a regional scale, vegetation condition parameters with high resolutions such as canopy clumping index (CI) and NDVI), can be obtained from remote sensing images, especially in the so-called “no man’s land” in the QTP (Sun et al., 2020). Furthermore, evaluating the status and dynamic changes in the relationship between pressures, vegetation conditions and ESs in the QTP is of great significance in ecosystem conservation and management.

Based on the above, we explored the following hypotheses: (1) the relationship between pressures and ESs changes over time, and (2) vegetation condition plays an indirect role in the impact of pressures on ESs. To this end, we first assess the spatiotemporal variation in multiple pressures. We then analyze the spatial patterns, bundle zones and temporal variations in ESs. Finally, we identify correlations between pressure, vegetation conditions and ESs, and propose corresponding ecosystem management and ecological conservation strategies.

## 2 Materials and methods

### 2.1 Study area

**Figure 1:**
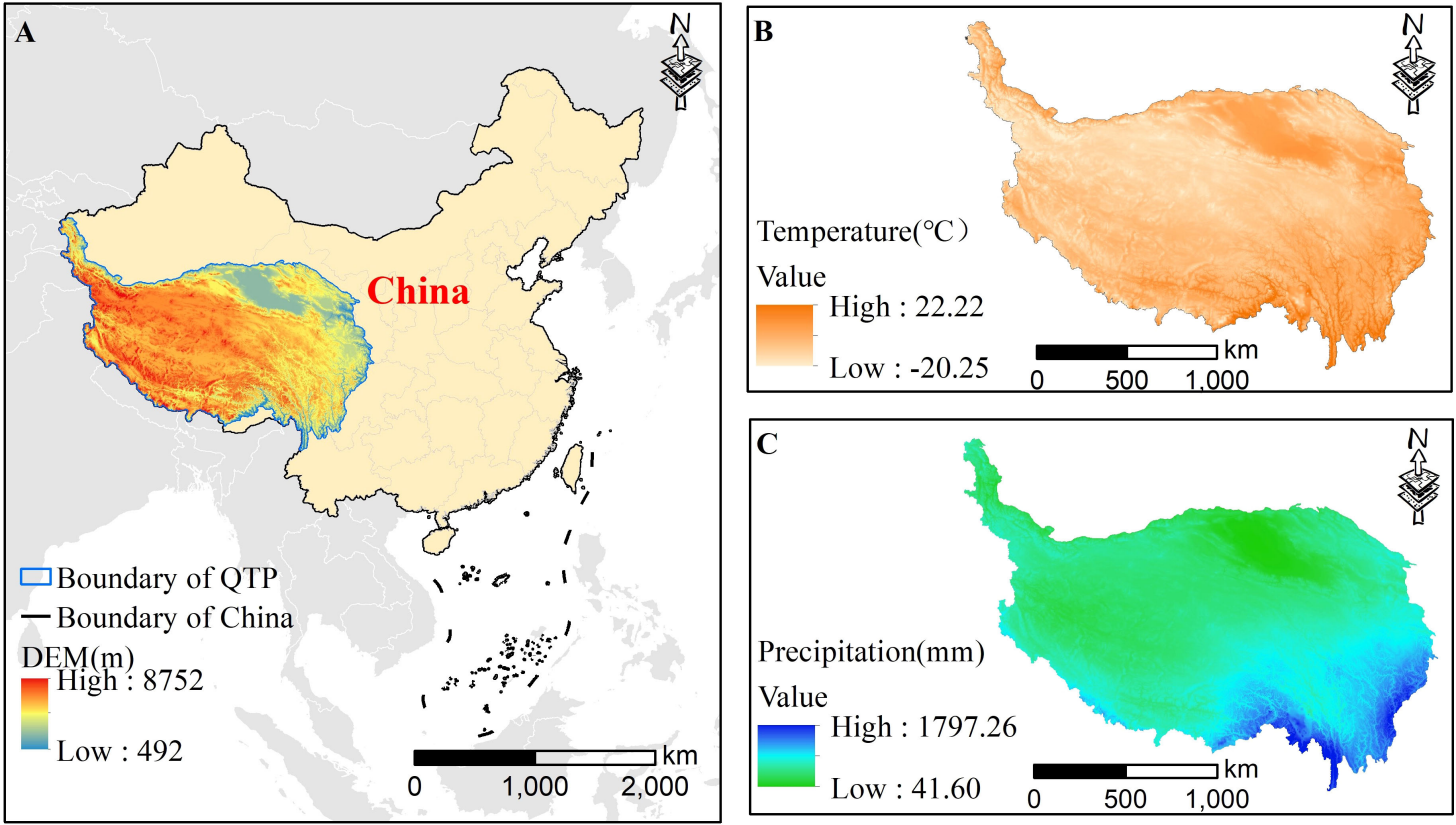
The location **(A)**, annual mean temperature **(B)** and annual mean precipitation **(C)** of the QTP.

The QTP is often called “the Roof of the World”, “the Asian water tower”, and “the Third Pole”. Located in Southwest China, the QTP is the highest plateau in the world, where the elevations reach 4000 m, and the QTP extends over 2.5 million km^2^ (Li et al., 2018) (**Figure 1**). The QTP is approximately 2800 km long, and the north and south are approximately 300 ~ 1500 km wide. The annual mean temperature and precipitation are 1.61°C and 413.6 mm, respectively. The QTP provides a large but vulnerable habitat for countless precious species (Xu et al., 2017). The QTP plays an essential role as a shelter for ecological security and is also the foundation for highland species husbandry.

### 2.2 Data sources

In this study, we used land use/cover datasets, road network datasets, DEM, meteorological datasets (i.e., temperature and precipitation), and vegetation condition datasets (i.e., NDVI, and CI)). All datasets are from 2000, 2010 and 2019, and are shown in **Table 1**.

**Table 1:** Principal data sources in the study.

No.	Category	Data source
1	Land use/cover datasets	Data Center for Resources and Environmental Sciences, Chinese Academy of Sciences (http://www.resdc.cn)
2	DEM	Geospatial Data Cloud Site, Computer Network Information Center, Chinese Academy of Sciences (http://www.gscloud.cn)
3	Road network datasets	Geographic Data Sharing Infrastructure, College of Urban and Environmental Science, Peking University (http://geodata.pku.edu.cn); OpenStreetMap (OSM) (https://download.geofabrik.de)
4	Meteorological datasets	China Meteorological Administration (http://data.cma.cn)
5	NDVI	Geospatial Data Cloud Site, Computer Network Information Center, Chinese Academy of Sciences (http://www.gscloud.cn)
6	CI	National Earth System Science Data Sharing Infrastructure (http://www.geodata.cn)

The datasets were aggregated using ArcGIS Desktop 10.3 (ESRI, USA). All datasets for pressures, vegetation conditions and ESs were normalized based on the min-max method to enable harmonization of inputs (Liu et al., 2020). In this study, the ESs and multiple pressures for each year were mapped using ArcGIS to show spatial variability over time.

### 2.3 Quantifying pressures of ESs

(1) Human activities

Human activities have been proven to have effects on the sustainable provision of ESs (Han et al., 2020). In this study, the distances from settlements and roads calculated by ArcGIS were used as metrics of human activities. These metrics represent the degree of human activities in the ecosystem to a certain extent (Sun et al., 2020).

(2) Meteorological pressures

Meteorological pressures affect ESs through temperature and precipitation (van der Geest et al., 2019; Vigl et al., 2021). Annual mean precipitation, annual maximum temperature, annual mean temperature and annual minimum temperature in 2000, 2010 and 2019 were interpolated using the Kriging interpolation method in ArcGIS.

(3) Landscape fragmentation

Fragmentation weakens the resilience of ecosystems (Vigl et al., 2021). Landscape fragmentation analysis was performed using Fragstats 4.2 (Kevin McGarigal, University of Massachusetts Amherst, USA). Landscape metrics are a description of landscape patterns and are often used to indicate the correlation between landscape change processes and landscape patterns (Plexida et al., 2014; Hu et al., 2021). Based on the meaning of the metrics and the ability to depict landscape fragmentation, we selected three landscape metrics for the class level, including Shannon’s diversity index (SHDI), largest patch index (LPI) and edge density (ED) (Mairota et al., 2013). We first extracted grassland, forestland, cropland and wetland from the land use/cover maps. The landscape metrics were then calculated based on the extracted data to more accurately analyze the impact of landscape fragmentation on vegetation conditions and ESs. **Table 2** shows the meaning of each metric.

**Table 2:** Landscape metrics and meanings.

Landscape metrics	Explanation
LPI	The proportion of the largest patch of a category in a landscape.
ED	The length of the edge per unit area in the landscape.
SHDI	SHDI equals the negative value of the sum of the area ratios of each patch type multiplied by the natural logarithm of its value.

### 2.4 Quantifying vegetation condition

The CI and NDVI were chosen as the indices for vegetation condition. The CI is important in the determination of hydrological processes, photosynthesis, and canopy radiative transfer (Fang, 2021), which can effectively represent vegetation conditions. The CI dataset is a time series CI product produced using sun angle, remote sensing images, and the optimal BRDF model, and completed with snow removal. NDVI can effectively characterize vegetation coverage and biomass, and was obtained from the vegetation instrument of remote sensing satellites (Sun et al., 2020).

### 2.5 Specification of ESs

We specified five ESs for the QTP on a scale of 1 km. Five ES indicators (soil retention, water yield, carbon sequestration, habitat quality – all regulating and landscape aesthetics – cultural) for 2000, 2010 and 2019 were chosen based on the Millennium Ecosystem Assessment (MA, 2005), data availability, and importance to the QTP. The weighted sum of ESs was used to obtain the cumulative ES value.

(1) Water yield

Freshwater provision is a crucial ES that is of great significance to human society (Zhao et al., 2018; Liu et al., 2019b). Moreover, water yield is also a fundamental basis for ecosystems. In this study, InVEST 3.9.2 (Stanford University, USA) was used to simulate the annual water yield. The model simulates the annual water yield in each watershed of the targeted area, using meteorological variables, biophysical variables, land use/cover data, terrain variables, and water consumption indices (Liu et al., 2019b). The model is derived from the Budyko curve. Parameter estimation in this model refers to previous literature (Zhang et al., 2007; Zhao et al., 2018).

(2) Soil retention

The sediment delivery ratio (SDR) module in InVEST was employed to simulate soil retention. The parameters needed for the run of this model include land use/cover data, erodibility data, terrain data, biophysical table, watershed division data, and threshold flow accumulation (Liu et al., 2019b). The model simulates the annual soil loss on each pixel based on the revised universal soil loss equation (RUSLE). The parameter estimation refers to previous references (Auerswald et al., 2014; Zhu, 2015).

(3) Carbon sequestration

NPP was chosen to indicate carbon sequestration, which is an essential component of the terrestrial carbon cycle. Carbon sequestration represents the release of carbon from terrestrial ecosystems, thereby conveying the current rate of increase in atmospheric CO_2_ (Jiang et al., 2018). In this research, NPP was simulated from MODIS images (250 m), using the Carnegie-Ames-Stanford Approach (CASA) (Potter et al., 1993; Tian et al., 2016; Liu et al., 2019b). The method refers to previous references (Mohamed et al., 2004; Zhu et al., 2007).

(4) Habitat quality

An ecosystem’s habitat quality refers to its capability to provide wildlife or specific populations with the necessary resources and conditions to survive (Terrado et al., 2016). The habitat quality module of InVEST was used to quantify the habitat quality based on habitat threat data and land use/cover map. The habitat quality maps were acquired for 2000, 2010 and 2019. The parameter estimation refers to a previous reference (Sharp et al., 2018).

(5) Landscape aesthetics

The scenic views of landscapes contribute to local communities’ well-being in a variety of ways. To increase local economies, landscape aesthetics play an essential role in attracting visitors. In the study, the scenic quality module (SQ) of InVEST was used to assess landscape aesthetics. The SQ calculates the value of the impacted visibility. The parameters needed to run the model are described in detail in the reference (Sharp et al., 2018).

### 2.6 Statistical analysis

#### 2.6.1 Identification of the heterogeneity of pressures

A Getis-Ord (Gi^*^) hot spot analysis in ArcGIS was used to identify clusters where z-scores were high (hot spots) and low (cold spots). With hot spot analysis, local patterns of spatial associations can be easily identified, making it more suited for comparing and visualizing pressure patterns (Getis and Ord, 1992). The Gi^*^ index is estimated as:


(1) $$G_{i}^{*}\left(d\right)=\frac{\sum_{j=1}^{n}w_{ij}x_{j}-\bar{x}\sum_{j=1}^{n}w_{ij}}{s\sqrt{\left\{\left[\left(\sum_{j=1}^{n}w_{ij}^{2}\right)-{\left(\sum_{j=1}^{n}w_{ij}\right)}^{2}\right]/\left(n-1\right)\right\}}}$$

where *w_ij_* is the scale distance, *x_j_* is the value of pressures of grid *j*, *x* is the average value of pressures, and *n* is the grid number. This was followed by the development of a cluster map that indicates the spatial patterns (Han et al., 2020).

#### 2.6.2 ES bundle identification

In this study, to identify the ES bundles for 2000, 2010, and 2019, the K-means cluster method was employed, where the optimal bundle number was determined with the help of NbClust package (Charrad et al., 2014). ES bundles were represented by radar diagrams, which were dimensionless, as they were derived from normalized ES values.

#### 2.6.3 Unravelling the relationship between pressures, vegetation conditions and ESs

**Figure 2:**
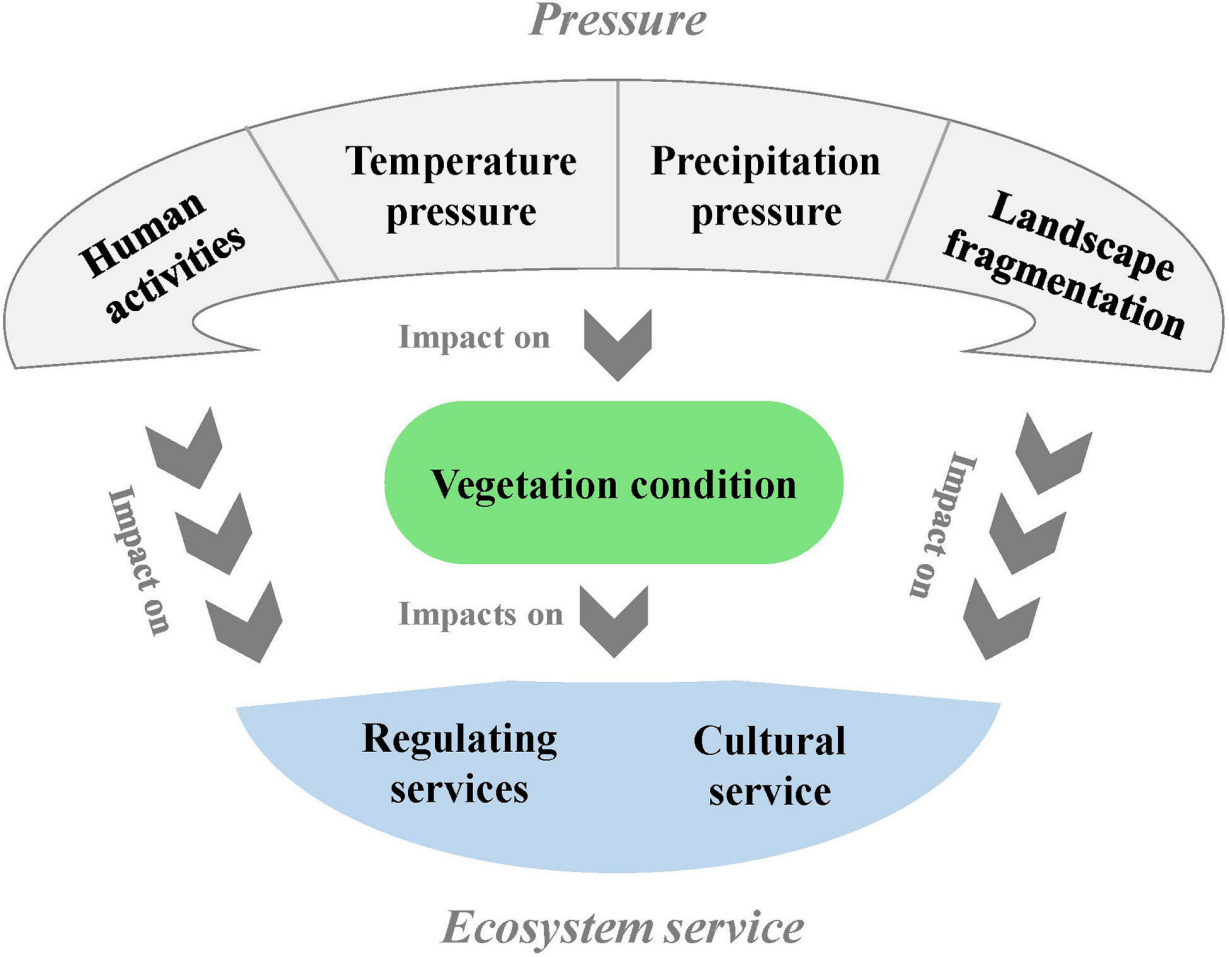
A conceptual SEM showing the possible relationship between pressure, vegetation condition and ESs.

We explored the relationship between pressures, vegetation conditions and ESs using structural equation modeling (SEM). By using SEM, we can implement conceptual models and estimate the relationships between their variables, where some latent variables are measured by manifest variables linked by linear regressions (Santos-Martin et al., 2013). A model was developed to indicate possible hypotheses about latent variables’ relationships (i.e., human activities, temperature, precipitation, landscape fragmentation, vegetation condition, regulating ESs and cultural ES) in a path diagram (**Figure 2**). Path analysis is one of the most commonly used methods in hypothesizing models, which can test whether the hypothesized causations can be verified (Qiu and Peng, 2022). We calculated and reported the standardized parameter values in the final SEM. The fitness of the SEM was checked using the adjusted goodness of fit index (AGFI), goodness of fit index (GFI), relative fit index (RFI), root mean square error of approximation (RMSEA), and Tucker−Lewis index (TLI). The SEM analysis was performed in SPSS AMOS 24 (IBM, USA). Statistical significance was set to *p* < 0.05.

## 3 Results

### 3.1 Spatiotemporal variation in anthropogenic and environmental pressures

**Figure 3:**
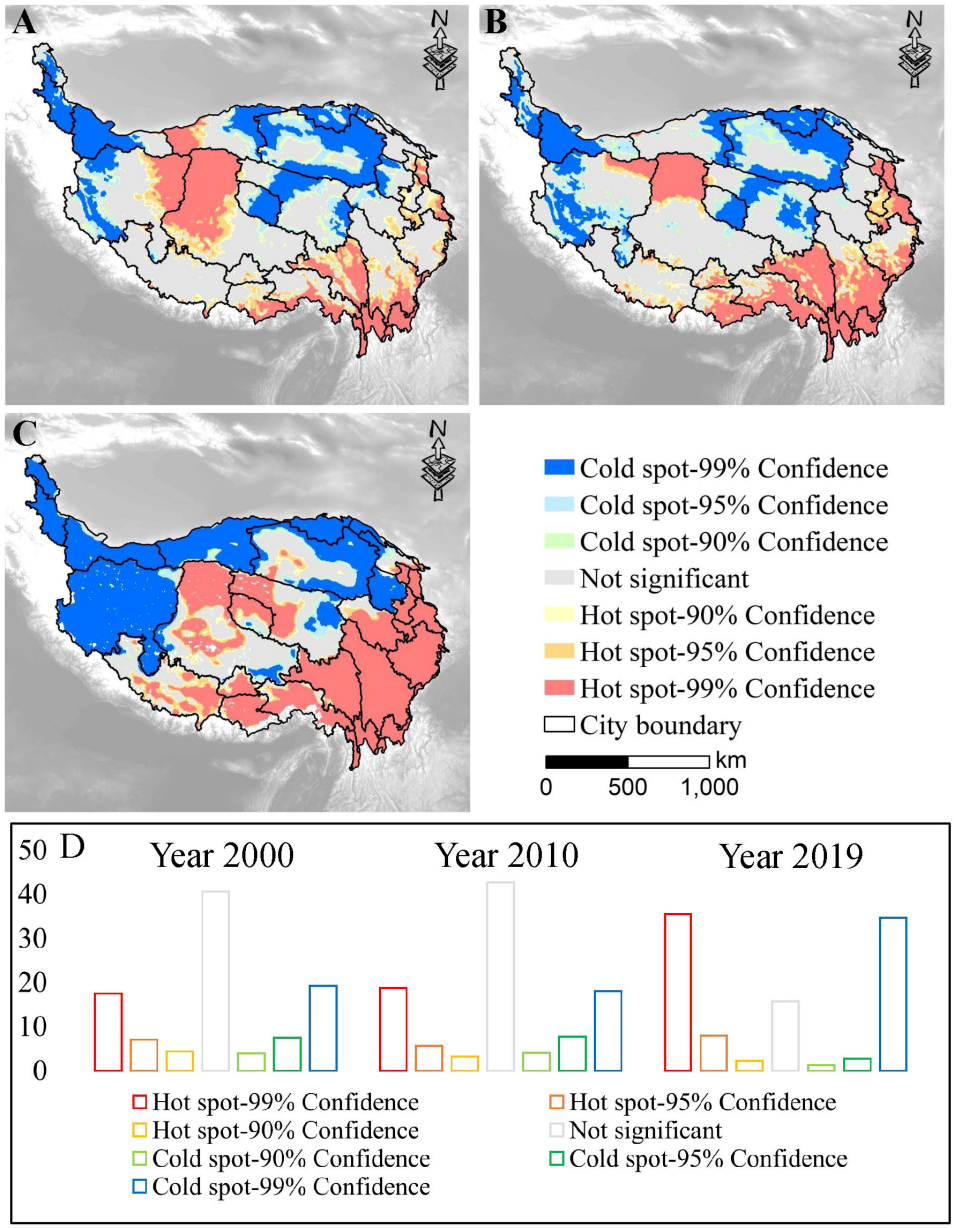
Gi^*^ scores of pressures in 2000 **(A)**, 2010 **(B)**, 2019 **(C)** and the proportions **(D)**.

The spatiotemporal variation in pressures was identified by hot spot analysis. The three heterogeneous units shown in **Figures 3A, B and C** were hot spots, cold spots and random spots, respectively. Hot spots accounted for 28.88%, 27.59%, and 45.66%, respectively for the three years. The proportion of cold spots was 30.51%, 29.79% and 38.60%, respectively for the three years, showing first a decrease and then an increase (**Figure 3D**). The proportion of random spots was 40.62%, 42.62%, and 15.74%, respectively, showing a slight rise and then a sharp decline. Spatially, heterogeneity is readily observed. For 2000, two large areas of hot spots were in the Midwest and southeast (**Figure 3A**). In the QTP, cold spots mostly occurred in the northwest, north, and center. Compared with 2000, hot spots in the Midwest shrank in 2010, but expanded in the southeast (**Figure 3B**). The cold spots in the west also expanded from 2000 to 2010. **Figure 3C** illustrates the largest cold and hot spot areas observed in 2019. Compared to the previous two periods, the cold and hot spots in 2019 showed a more pronounced spatial agglomeration. That is, from northwest to southeast, there were cold spots, cold-hot spot intersections and hot spots regions.

### 3.2 Spatiotemporal variation in ESs

#### 3.2.1 Cumulative ESs

**Figure 4:**
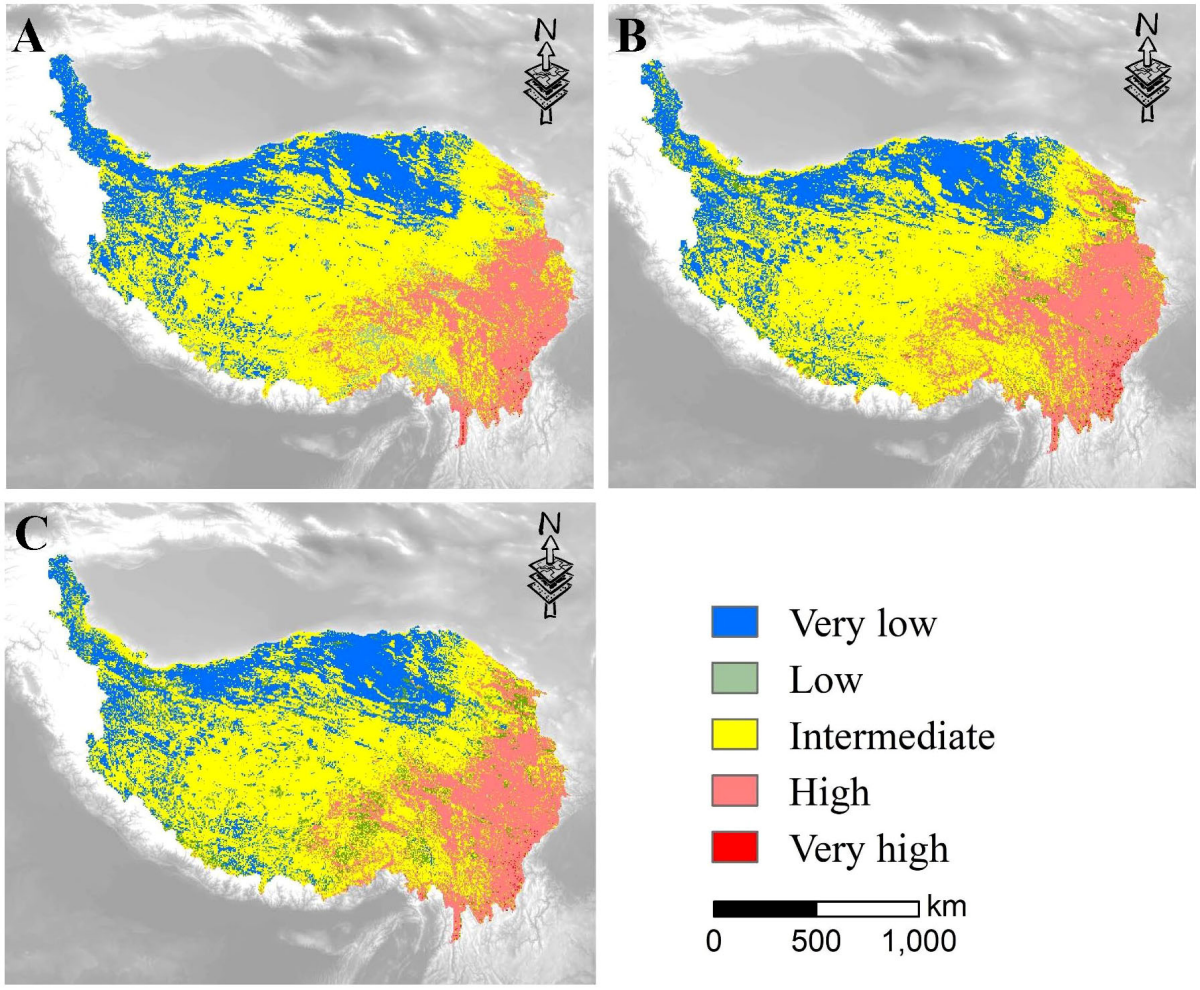
Cumulative ES provision in the QTP for 2000 **(A)**, 2010 **(B)** and 2019 **(C)**.

For the five ESs evaluated in the study, the cumulative value of ESs registered a maximum score of 2.9 (**Figure 4**). We divided the ES values into five categories, namely very low, low, intermediate, high and very high, corresponding to the level of cumulative value of ESs based on the natural break method in ArcGIS. The areas with high and very high cumulative ESs were located in the southeastern part of the QTP, comprising 17.44%, 20.64%, and 19.14% of the total area in 2000, 2010 and 2019, respectively. The areas with low and very low values were located in the north, west and northwest regions, with 27.32%, 25.71%, and 29.07% respectively for 2000, 2010 and 2019. The area with intermediate values showed a downward trend from 2000 to 2019. Overall, the ES supply in the QTP showed a gradual increase from northwest to southeast during all three periods studied.

#### 3.2.2 ES bundle mapping

**Figure 5:**
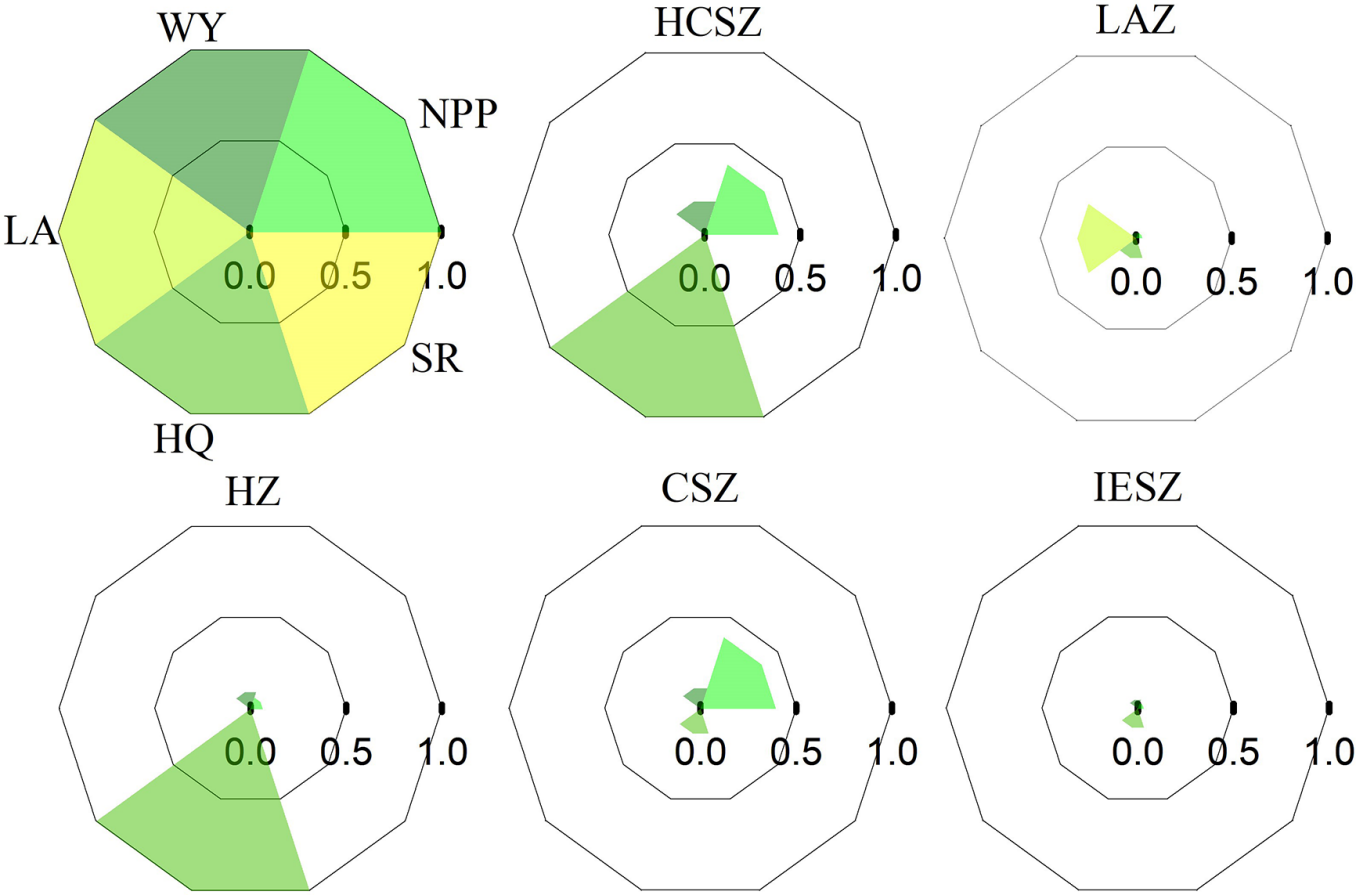
Five ES bundles in the QTP from 2000 to 2019. WY, water yield; SR, soil retention; HQ, habitat quality; LA, landscape aesthetics.

The grids of the QTP were divided into 5 bundles, where grids with similar ESs were bundled together. According to the supply of ESs, five bundles were named: “Habitat and carbon sequestration zone (HCSZ)”, “Landscape aesthetics zone (LAZ)”, “Habitat zone (HZ)”, “Carbon sequestration zone (CSZ)”, and “Insufficient ES zone (IESZ)”. The ES bundles are visualized in **Figure 5**.

HCSZ was characterized by high NPP and habitat quality with a low delivery of soil retention, landscape aesthetics and water yield. In LAZ, landscape aesthetics were high, but NPP, water yield, habitat quality and soil retention were low. In HZ, habitat quality was high, but NPP, water yield, landscape aesthetics and soil retention were low. In CSZ, NPP was high, but water yield, landscape aesthetics, habitat quality and soil retention were low. In IESZ, all five ESs were low.

**Figure 6:**
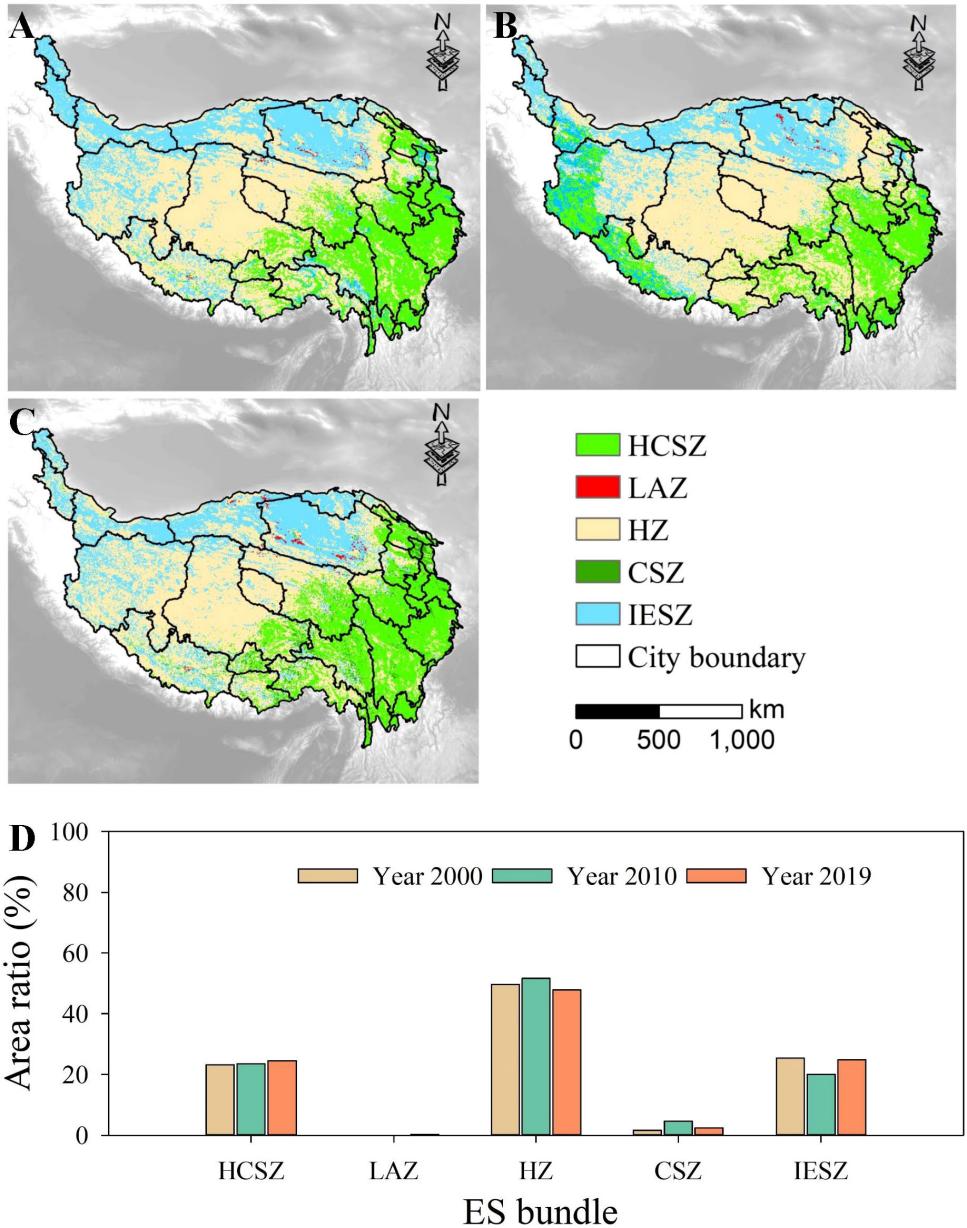
ES bundles in 2000 **(A)**, 2010 **(B)**, and 2019 **(C)** and dynamics over time **(D)**.

**Figure 6** shows the spatial distribution of ES bundles and their temporal dynamics. The area of HZ in 2000, 2010 and 2019 accounted for 49.7%, 51.6%, and 47.8% respectively, making it the largest group. In addition, the IESZ was another large area group, with an area share of 25.4%, 20.1%, and 24.8% in 2000, 2010 and 2019 respectively. The area of the LAZ was 0.17%, 0.16%, and 0.34% in 2000, 2010 and 2019 respectively, making the LAZ the smallest ES bundle. ES bundles showed obvious spatial agglomeration during the study period. For example, girds belonging to HCSZ were mostly located in the eastern and southeastern regions during this period, and also in the western regions in 2010. The grids belonging to the IESZ were mostly located in the northern and northwestern regions, and the spatial pattern remained relatively stable during the whole study period. As the largest ES bundle in the QTP, the HZ occupied the highest fraction of the central region. From 2000 to 2019, the area of HCSZ, LAZ and CSZ all increased, with the LAZ increasing to the most extent (107%) but still accounting for the smallest proportion. However, both the HZ and IESZ areas fluctuated and decreased, with the HZ experiencing the largest decrease (3.6%).

### 3.3 The impacts of multiple anthropogenic and environmental pressures and vegetation conditions on ESs

**Figure 7:**
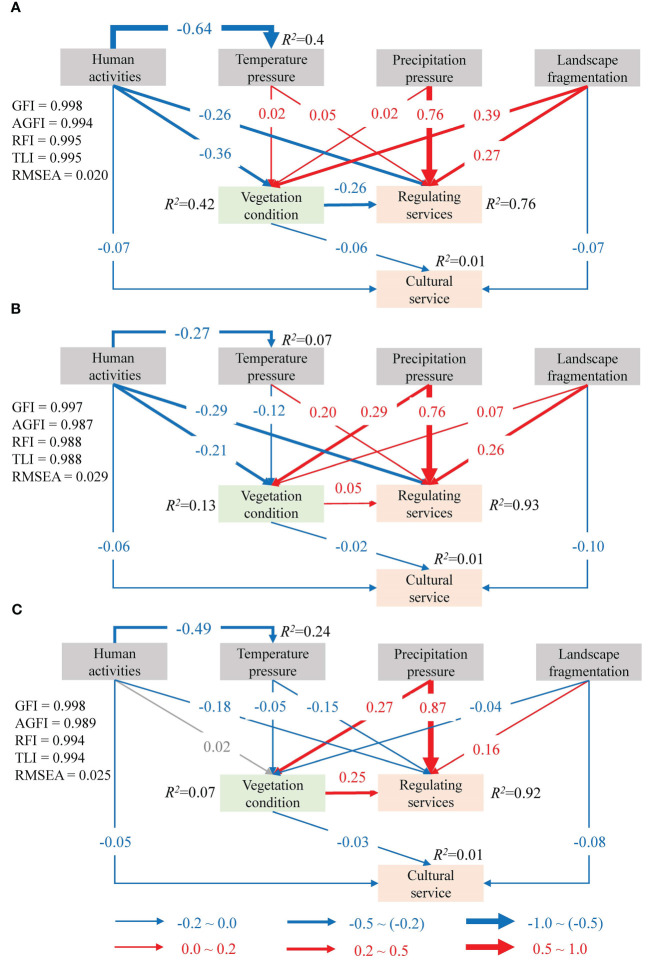
Impacts of pressures and vegetation condition on ESs in 2000 **(A)**, 2010 **(B)** and 2019 **(C)**. Blue and red arrows indicate significant negative and positive impacts (*p* ≤ 0.01), respectively; Grey arrow indicates that the relationship is not significant; *R^2^* indicates the proportion of variance explained.

The SEM results explained greater than three-fourths of the variance of regulating services (*R^2^* ≥ 0.76), and only 1% of the variance in cultural service (*R^2^* = 0.01) in all three years. Human activities, temperature, precipitation, and landscape fragmentation together explained 42%, 13%, and 7% of the variability in vegetation conditions in 2000, 2010 and 2019, respectively. All the components analyzed in the SEM are strongly related, except for the relationship between human activities and vegetation conditions in 2010 (**Figure 7C**). The partial effects (rα) of precipitation on regulating services were the largest and significantly positive (*rα* ≥ 0.76, *p* < 0.001). Landscape fragmentation had the largest effect on cultural service (-0.10 ≤ *rα* ≤ -0.07). Vegetation conditions had weak negative effects on cultural service (*rα* = -0.06, -0.02, and -0.03 for 2000, 2010, and 2019, respectively). Additionally, vegetation conditions negatively affected regulating services in 2000 (*rα* = -0.26), while there was a positive relationship between vegetation conditions and regulating services in both 2010 and 2019 (*rα* = 0.05 and *rα* = 0.25, respectively).

In 2000, temperature, precipitation and landscape fragmentation had positive and significant effects on vegetation conditions and regulating services (**Figure 7A**). The opposite was found for human activities which were significantly and negatively associated with vegetation conditions, regulating services and cultural service. In 2010, temperature, precipitation, landscape fragmentation, and vegetation conditions had positive effects on regulating services (**Figure 7B**). In contrast, human activities had negative effects on vegetation conditions, regulating services, and cultural service. In 2019, precipitation, landscape fragmentation, and vegetation condition had significant and positive effects on regulating services, while human activities and temperature had negative effects (**Figure 7C**).

## 4 Discussion

### 4.1 Spatiotemporal variations in ESs and pressures

This study showed that ESs and associated multiple pressures had spatiotemporal variations, which is consistent with previous studies (Mohamed et al., 2004; Liu et al., 2019b; Ma et al., 2021; Vigl et al., 2021). Specifically, the Gi^*^ analysis showed that the hot spots of pressures were mainly located in the south, southeast and mid-western regions of the QTP, which indicated a trend of increasing fluctuation from 2000 to 2019 (**Figure 3**). This is proven to be consistent with previous studies on precipitation, temperature, and human activity intensity pressures (Sun et al., 2020; Hou et al., 2021; Ma et al., 2021). Moreover, the results also demonstrated that the pressure distribution throughout the QTP was extremely inhomogeneous in space and constantly changing over time (**Figure 3**). This pattern may be caused by a complex mechanism of interaction between water, heat, soil, animals, and the decision-making of managers over the vast area of the QTP (Martin et al., 2009; Liu et al., 2019a; Ma et al., 2021).

From 2000 to 2019, the regions with high and very high cumulative ESs showed an increase, but the increase area was slightly less than the regions with low and very low cumulative ESs (**Figure 4**). This suggests that one third of the QTP area has a low supply of ESs, which might bring about the urgency of regional ecosystem conservation and management (Liu et al., 2020). Additionally, from the perspective of the spatial pattern, the ES supply in the QTP showed a trend of gradual increase from northwest to southeast (**Figure 4**). ESs were interwoven with multiple pressures, that is, areas with high ES supply were often hot spots of pressures, while areas with low ES supply levels were often cold spots of pressures, which was particularly obvious in the southeast and northwest of the QTP (**Figures 3**, **4**). This finding was consistent with other studies of the QTP (Sun et al., 2020; Hou et al., 2021), but contradicted those of Alp and Spain (Santos-Martin et al., 2013; Vigl et al., 2021). The possible reason is that the ESs in the QTP were affected by pressures at relatively low levels compared to other regions of the world (The State Council Information Office of the People’s Republic of China, 2018). Therefore, when evaluating ESs and multiple pressures, attention should be given to their complex spatiotemporal variations across different regions, thus supporting ecosystem management.

### 4.2 The impact pathways of pressure and vegetation condition on ESs

Pressures may affect different ESs with different intensities (Vigl et al., 2021). Our results demonstrated that multiple pressures had significant and varying degrees of influence on ESs (**Figure 7**). Previous studies mostly focused on the direct impact of pressures on ESs, and less attention was given to the intermediate factors between the two, which made it difficult to fully reveal the impact mechanism of the pressures on ESs (Liu et al., 2019b; van der Geest et al., 2019; Sun et al., 2020). Our study indicated that the pressures influenced ESs in both direct and indirect ways (**Figure 7**). Also, the impact pathway analysis showed that the normalized total effects of precipitation and landscape fragmentation on the regulating services were positive and largest, greater than 0.75 and 0.16, respectively, over the entire study period. For the cultural service, landscape fragmentation and human activities had strong negative total effects, below -0.07 and -0.05, respectively. To achieve effective ecosystem conservation planning that seeks to maintain and sustain ES supply, impact pathways of pressures on ESs should be identified to improve corresponding management measures.

(1) The first pathway in which pressures influenced ESs was the direct pathway, namely the pressure-ES pathway (PESP) (**Figure 7**). Human activities had a significant negative influence on regulating services, while precipitation and landscape fragmentation had a significant positive impact. These results were consistent with those of previous researches (Sun et al., 2020; Chen et al., 2021; Vigl et al., 2021). The effect of temperature pressure on regulating services changed at different times, namely, it was positive in 2000 and 2010 and negative in 2019. One possible reason was that temperatures in 2019 were significantly cooler than those in 2000 and 2010, with the lowest annual mean temperature in the study period. In addition, both human activities and landscape fragmentation negatively affected cultural service. Since cultural service was represented by landscape aesthetics in this study, the above findings suggested that when human activities and landscape fragmentation intensified, they somehow hindered the provision and accessibility of vision, resulting in a decrease in the cultural service (Vigl et al., 2021; Li, 2022).

(2) The second pathway considered vegetation conditions, namely, the pressure-vegetation-ES pathway (PVESP). The indirect pathway analysis indicated that multiple pressures could change the supply of ESs by influencing vegetation conditions (**Figure 7**). This also verified the hypothesis of this study that vegetation played a certain role in the pressure-ES relationship (Chen et al., 2021). Previous studies also found that vegetation index was related to regulating services (Lü et al., 2012; Feng et al., 2017). Li (2022) studied ES supply and demand and found that ES balance was influenced by vegetation conditions. The effect of PVESP was small, indicating that the effect of multiple pressures on ESs through the influence of vegetation is generally smaller than the effect of pressures on ESs directly. This may also be the reason why many studies rarely consider vegetation factors when assessing the relationship between pressures and ESs. However, the impact of vegetation conditions on ES supply may vary with the size of vegetation cover. For example, studies of dune ecosystems have shown that increasing forest area improve the supply of regulating services (Dang et al., 2021). Therefore, the role of vegetation in the relationship between pressures and ESs should be emphasized in future studies.

### 4.3 Ecosystem management implications in the QTP and beyond

In this study, we found that both ESs and multiple pressures had spatiotemporal heterogeneity (**Figure 3**, **4**). This suggests that policy-makers should consider the dynamic situation when formulating ecosystem conservation planning, rather than follow the research results at a certain time (Ma et al., 2021; Pyles Marcela et al., 2022). There are two sides to effective ecosystem management, namely, the sustainable supply of ESs and how to cope with multiple pressures (Vigl et al., 2021). Given more extensive and in-depth ES assessments and greater awareness in response to pressures, there is a good opportunity to improve the adequacy of ecological management (Guo et al., 2020; Mandle et al., 2020; Meacham et al., 2022). In particular, it should be noted that the pressures in the QTP gradually showed a trend of increasing aggregation, especially in the eastern and southeastern regions (**Figure 3**) (Sun et al., 2020). In these areas, where road construction and urbanization were increasing, land use optimization and ecological restoration should be used to strengthen regulation and reduce the negative impact of human activities (Lü et al., 2012; Lü et al., 2021). The climatic influence on the ESs was also analyzed in the research, and the findings indicated that precipitation was more highly correlated with regulating services (**Figure 7**). Precipitation was proven to increase in the northeast and west, while it decreased in the southeast (Chen et al., 2021). Furthermore, precipitation is an important source for soil moisture recharge of the QTP, making it crucial to vegetation and ecosystems (Chen et al., 2021). Therefore, management measures that consider precipitation status and resource utilization are crucial ways to mitigate the negative influence of future climate change in areas with reduced precipitation (Liu et al., 2019b).

Considering the needs of ecological conservation, determining how to assess the relationship among pressures, vegetation, and ESs has become a crucial issue globally (Hou et al., 2021). The complex relationships between ESs render it difficult to promote different ESs simultaneously with the same management measures (Lü et al., 2012). In this study, we clustered the ESs into five ES bundles according to their supply level (**Figure 6**), to facilitate ecological conservation and management in targeted areas. At this juncture, the integration of ES bundles and pressure patterns can provide a basis for scientific decision-making (Yu et al., 2021). For example, the HCSZ was characterized by providing NPP and habitat, and the pressure had been increasing over time in this region (**Figure 4**). Also, the HCSZ was positively affected by precipitation and landscape fragmentation, negatively affected by human activities, and alternately affected by temperature pressure and vegetation conditions. Therefore, the impact of human activities should be reasonably controlled in this area through scientific city planning (Sun et al., 2019; Sun et al., 2020). Furthermore, optimizing the regional vegetation pattern may benefit the maintenance and enhancement of ESs (Yapp et al., 2010). As precipitation affected the regulating services most (Chen et al., 2021), the efficient utilization of precipitation resources may help improve vegetation conditions, thereby ensuring the continuous supply of ESs (Liu et al., 2019b). As another example, the supply of multiple ESs in the IESZ was relatively poor. Most of the IESZ faced less pressures (**Figure 4**), and the land use types were mainly glaciers, bare rocks, Gobi and deserts. In the ecosystem management of the region, coping with the pressures was not an important component, but rather the focus should be on how to exploit the vegetation resources and maintain the supply of one or several ESs to improve the overall ES supply level of the QTP.

This study focuses on pressures and ESs and follows “spatiotemporal variation analysis, impact pathway identification, and ecosystem management support”, which has a good reference for the use of ES research to guide regional management in other alpine plateau regions. In addition, most of the data in this study are available through remote sensing and field sampling, making it easier to focus on spatial patterns and temporal changes in ESs and pressures, and thus better support ecosystem management decisions. The study primarily indicates rankings of ESs and pressures, rather than exact values, which can be easily performed in study of multiscale pressure-ES relationships and ecosystem management.

Our assessment of ESs was mainly based on InVEST model, which was not compared with field site data. The unverified calibration of the simulation may lead to some uncertainty in the results of the models. However, it is difficult to verify the model with field data in such a large area as the QTP under current conditions. We will strengthen this in future studies to make the results more accurate and support regional ecological management.

## 5 Conclusion

Ecosystem management is challenged by multiple pressures. Synthesizing and learning pathways of “pressure-ES” and “pressure-vegetation-ES” is necessary to deepen the understanding of the impact mechanism of pressures on ESs and how they are used for landscape optimization and ecosystem conservation. Our study indicated that both spatial agglomeration and the amount of pressures on ESs increased in the QTP from 2000 to 2019. Although regions with cumulative ESs above the intermediate level accounted for 70% of the QTP with spatial heterogeneity, regions with poor ES supply were growing rapidly. Moreover, the results highlight the evidence that both PESP (direct) and PVESP (indirect) were vital pathways, providing a different perspective to dissect how pressures affect ESs. Our findings emphasize the need to consider the complex interactions between pressures, vegetation, and ESs in ecosystem conservation and management practices. Improvements in spatially targeted policy design are expected in the future by identifying ES bundles and the relative importance of multiple pressures affecting vegetation and ESs.

## Data availability statement

The original contributions presented in the study are included in the article/supplementary material. Further inquiries can be directed to the corresponding author.

## Author contributions

YXL: writing, reviewing, editing, and validation. YHL: conceptualization, editing, and resources. MZ: data curation, writing, and methodology. BF: conceptualization and reviewing. All authors contributed to the article and approved the submitted version.
